# Optimal practices for the management of hereditary transthyretin amyloidosis: real-world experience from Japan, Brazil, and Portugal

**DOI:** 10.1186/s13023-023-02910-3

**Published:** 2023-10-12

**Authors:** Yukio Ando, Marcia Waddington-Cruz, Yoshiki Sekijima, Haruki Koike, Mitsuharu Ueda, Hiroaki Konishi, Tomonori Ishii, Teresa Coelho

**Affiliations:** 1 Department of Amyloidosis Research, Faculty of Pharmaceutical Sciences, Nagasaki International University, 2825-7 Huis Ten Bosch Machi, Sasebo City, Nagasaki 859-3298 Japan; 2 Hospital Universitário Clementino Fraga Filho, Centro de Estudos em Paramiloidose Antônio Rodrigues de Mello, Universidade Federal do Rio de Janeiro, Rio de Janeiro, RJ Brazil; 3 Department of Medicine (Neurology and Rheumatology), Shinshu University School of Medicine, Matsumoto, Japan; 4 Department of Neurology, Nagoya University Graduate School of Medicine, Nagoya, Japan; 5 Present Address: Division of Neurology, Department of Internal Medicine, Faculty of Medicine, Saga University, Saga, Japan; 6 Department of Neurology, Graduate School of Medical Sciences, Kumamoto University, Kumamoto, Japan; 7 Pfizer Japan Inc., Tokyo, Japan; 8 Andrade’s Center for Familial Amyloidosis, Hospital Santo António, Centro Hospitalar Universitário Do Porto, Porto, Portugal

**Keywords:** Asymptomatic, ATTR amyloidosis, Biopsy, Cardiomyopathy, Gene mutation carrier, Genetic counseling, Neuropathy, Noninvasive testing, Practical guidance, Predictive genetic testing

## Abstract

Hereditary transthyretin (ATTRv) amyloidosis is a rare and autosomal dominant disorder associated with mutations in the transthyretin gene. Patients present with diverse symptoms related to sensory, motor, and autonomic neuropathy, as well as gastrointestinal, ocular, cardiac, renal and orthopedic symptoms, resulting from the deposition of transthyretin amyloid fibrils in multiple organs. The progressive nature of ATTRv amyloidosis necessitates pre- and post-onset monitoring of the disease. This review article is primarily based on a collation of discussions from a medical advisory board meeting in August 2021. In this article, we summarize the best practices in amyloidosis centers in three major endemic countries for ATTRv amyloidosis (Japan, Brazil, and Portugal), where most patients carry the Val30Met mutation in the transthyretin gene and the patients’ genetic background was proven to be the same. The discussions highlighted the similarities and differences in the management of asymptomatic gene mutation carriers among the three countries in terms of the use of noninvasive tests and tissue biopsies and timing of starting the investigations. In addition, this article discusses a set of practical tests and examinations for monitoring disease progression applicable to neurologists working in diverse medical settings and generalizable in non-endemic countries and areas. This set of assessments consists of periodic (every 6 to 12 months) evaluations of patients’ nutritional status and autonomic, renal, cardiac, ophthalmologic, and neurological functions. Physical examinations and patient-reported outcome assessments should be also scheduled every 6 to 12 months. Programs for monitoring gene mutation carriers and robust referral networks can aid in appropriate patient management in pre- to post-onset stages. For pre- and post-symptom onset testing for ATTRv amyloidosis, various noninvasive techniques are available; however, their applicability differs depending on the medical setting in each country and region, and the optimal option should be selected in view of the clinical settings, medical environment, and available healthcare resources in each region.

## Background

Hereditary transthyretin (ATTRv) amyloidosis is an autosomal dominant disorder associated with mutations in the transthyretin (*TTR*) gene [1]. Pathogenic mutations in the *TTR* gene cause destabilization of transthyretin tetramers, resulting in the aggregation of insoluble amyloid fibrils and their deposition in multiple organs [1]. Patients with ATTRv amyloidosis exhibit a variety of symptoms associated with sensory, motor, and autonomic neuropathy [1, 2]. Gastrointestinal manifestations, ocular involvement, cardiac disorders, renal impairment, central nervous system manifestations, and carpal tunnel syndrome (CTS) are also common clinical symptoms [1–6].

Because ATTRv amyloidosis progresses slowly or moderately and several therapeutic options are available now, it is essential for attending physicians to capture the early signs and symptoms of disease onset [1, 7–10]. Family members of patients with ATTRv amyloidosis often have pathogenic mutations in the *TTR* gene and are at risk of developing the disease; thus, they are primary targets for monitoring early signs and symptoms [7, 11, 12]. Indeed, in the Transthyretin Amyloidosis Outcomes Survey (THAOS) Registry, more than one-third of asymptomatic *TTR* gene mutation carriers developed ATTRv amyloidosis within a median of approximately 2 years of enrolment [13]. Once a patient has been diagnosed with ATTRv amyloidosis, it is important to initiate treatment and continuously monitor disease progression and treatment response [13].

Amyloidosis experts worldwide have issued several recommendations for the management of asymptomatic *TTR* gene mutation carriers [7, 11, 12], as well as those for continuous monitoring of disease progression after disease onset [14, 15]. Additionally, in 2022, the International Society of Amyloidosis (ISA) issued guidelines for the treatment and monitoring of ATTRv amyloidosis, which include a minimum set of assessments to monitor disease progression [16]. Practical and convenient guidance for pre– and post–symptom onset testing for ATTRv amyloidosis that could benefit both amyloidosis specialists and general neurologists working in diverse medical settings would be useful.

This review article is based on the authors’ clinical experience and a collation of discussions from a medical advisory board meeting in August 2021. The meeting was joined by authors from Japan, Brazil, and Portugal and included physician neurologists from amyloidosis referral centers who routinely treat patients with ATTRv amyloidosis in collaboration with clinicians from other specialties. This review article was composed as an initiative to propose a practical approach for family testing and pre– and post–symptom onset testing for ATTRv amyloidosis. In this article, after summarizing the general overview of ATTRv amyloidosis, we discuss the follow-up strategy for *TTR* gene mutation carriers and diversity of methods used to capture the signs of disease onset. We also present the critical roles of referral centers and referral networks as well as a set of practical patient follow-up methods that can be implemented in various neurology practice settings. In addition, future perspectives in the management of ATTRv amyloidosis are highlighted.

Currently, the availability of equipment and tests to perform patient assessments differs across countries, regions, and medical institutions, and the medical procedures applied in routine practice are primarily based on each physician’s clinical experience. Published evidence from Japan, Brazil, and Portugal, the three endemic countries that share the same patient genetic background [17], is sufficient to discuss the optimal strategy for testing and monitoring of ATTRv amyloidosis; however, evidence derived from non-endemic countries remains lacking, which necessitates practical approaches generalizable to regions outside the endemic countries.

### ATTRv amyloidosis: general overview and epidemiology

The global number of patients with ATTRv amyloidosis was estimated to be 10,186 (range: 5526–38468) [18]. However, the evidence supporting this estimate is heterogeneous and carries a risk of bias, and the broad range of the estimated prevalence indicates the need for increased awareness regarding this rare disease among neurologists [18].

More than 140 different mutations in the *TTR* gene have been reported [19], and the primary symptoms of ATTRv amyloidosis may vary depending on the genetic mutations involved [1]. The most common mutation associated with the onset of ATTRv amyloidosis is Val30Met (p.Val50Met) [1, 20]. Patients with ATTRv amyloidosis carrying this mutation can be categorized into two groups: patients with early-onset disease (age < 50 years), who are more common in the endemic areas, and those with late-onset disease (age ≥ 50 years), who are also observed in non-endemic areas [21]. The late-onset disease can sometimes manifest as sporadic cases [22, 23]. According to the THAOS Registry, approximately 35%–40% of patients with ATTRv amyloidosis carrying the Val30Met mutation were classified as having late-onset disease [2, 24]. The severity of the initial symptoms has been reported to differ between late-onset and early-onset diseases [21, 24]. The penetrance of the Val30Met mutation is generally high in early-onset disease and low in late-onset disease [1], may be greater in cases of maternal inheritance and in men [25, 26], but varies greatly among families or individuals [26, 27]. Data for other mutations remain insufficient to establish the exact penetrance [28].

Several countries, such as Portugal, Brazil, Japan, and Sweden, have been historically considered endemic foci of the disease [10, 20]. In addition, cases have been reported in other countries, and non-Val30Met mutations have been often observed [2]. The characteristics of patients with ATTRv amyloidosis differ substantially among regions [29–40] (Table 1). However, these results should be interpreted with caution because the sample size varies widely among reports. Moreover, some publications are not based on nationwide surveillance, and thus, the number of patients assessed in each publication may be substantially lower than the total number of patients in each country or region.

**Table 1:** Characteristics of patients with ATTRv amyloidosis: examples from the published literature

Variable	Japan	Brazil	Portugal	Sweden (Norrbotten)	Spain (Majorca)	Greece (outside Crete)	Italy (Lazio)	France	US (Minnesota)	China	Taiwan	Singapore
Male sex, %	65.3	52.5	56.0	68.8	50.7	66.7	63.0	61.7	79.3	77.8	69.6	58.6
Early-onset patients, %	29.2	NA	71.3	8.6	NA	NA	17.0	26.7	NA	46.3	NA	17.2
Late-onset patients, %	61.2	NA^a^	28.7	91.4	NA	NA	83.0	73.3	NA	53.7	NA	82.8
Median age at onset or diagnosis, years	NA	32.5	38	72.8	NA	NA	NA	NA	59.6	NA	NA	57
Mean age at onset or diagnosis, years	NA	NA	42.8	70.4	49.8	51.1	NA	NA	NA	47.8	58.2	NA
Val30Met mutation, %	65.0^b^	91.9	NA	95.7	100	44.4	53.0	58.3	15.8	22.2	0	13.8
Non-Val30Met mutation, %	34.5^b^	8.1	NA	4.3	0	55.6	47.0	41.7	84.2	77.8	100	86.2
Positive family history of ATTRv amyloidosis, %	57.5	90.6	NA	61.3	69.3	44.4	58.0	60.0	47.7	56.4	79.7	75.9
References	[29]	[30]	[31]	[32]	[33]	[34]	[35]	[36]	[37]	[38]	[39]	[40]

The number of patients assessed in each publication (Japan, 219; Brazil, 160; Portugal, 500; Sweden [Norrbotten], 93; Spain [Majorca], 75; Greece [outside the island of Crete], 27; Italy [Lazio], 100; France, 60; US [Minnesota], 266; China, 54; Taiwan, 79; Singapore, 29) does not represent the total number of patients with ATTRv amyloidosis in each country or region

^a^According to the THAOS Registry data, among 137 patients with the Val30Met mutation in Brazil, 40 (29.2%) were late-onset patients [24].

^b^One patient (0.5%) underwent genetic testing, but mutation data were not available

*ATTRv* hereditary transthyretin, *NA* not available, *THAOS* Transthyretin Amyloidosis Outcomes Survey, *US* United States

### Follow-up methods for pre-symptomatic *TTR* gene mutation carriers

As *TTR* gene mutation carriers are at risk of developing ATTRv amyloidosis, monitoring of early signs and symptoms of disease onset in this population is crucial [7, 11, 12].

#### Need for a follow-up program for* TTR* gene mutation carriers

**Fig. 1:**
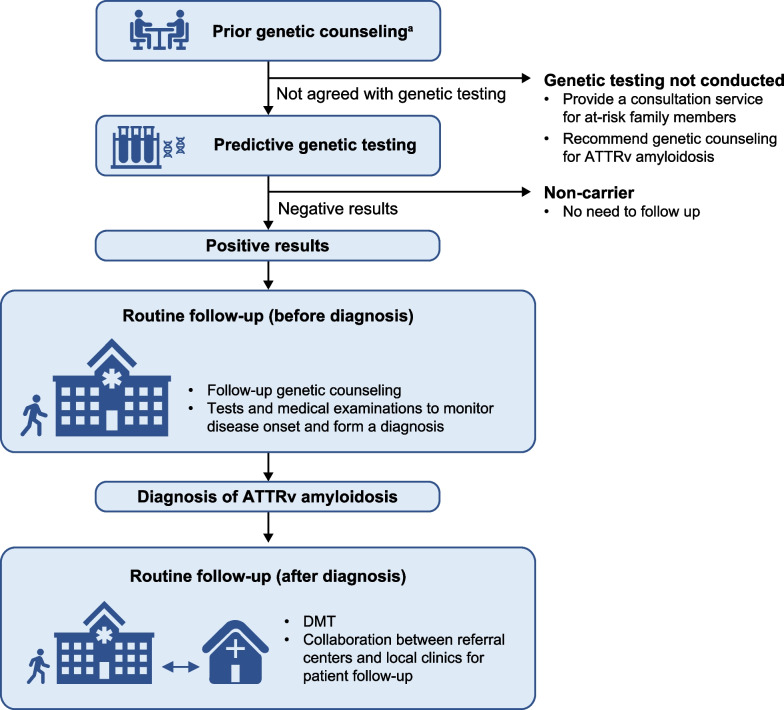
Follow-up program for the management of *TTR* gene mutation carriers. ^a^The prerequisites for offering predictive genetic testing should follow the local guidelines for genetic testing and diagnosis. *ATTRv* hereditary transthyretin, *DMT* disease-modifying treatment, *TTR* transthyretin

For the appropriate pre–symptom onset testing for *TTR* gene mutation carriers, a follow-up program consisting of genetic counseling, predictive genetic testing (for those who agree), and routine follow-up visits is crucial (Fig. 1).

Predictive genetic testing offered to family members of known disease carriers allows the determination of whether an individual has pathogenic *TTR* mutations and is at risk of developing ATTRv amyloidosis. Such individuals should receive genetic counseling in advance and be fully informed about the risks and benefits of undergoing genetic testing. The decision to offer predictive genetic testing to individuals who have difficulty making an autonomous decision (e.g., minors) must be made based on local guidelines for genetic testing and diagnosis. Those who have tested positive for *TTR* gene mutations should be offered periodic follow-up genetic counseling sessions to increase their awareness of the disease. In addition, follow-up visits to enable the earliest detection of disease onset should be scheduled [7, 12, 27, 41].

#### Differences in the follow-up methods between Japan, Brazil, and Portugal

Amyloidosis experts have proposed optimal management strategies for asymptomatic *TTR* gene mutation carriers [7, 11, 12]; however, clinical practice for carrier management often differs between countries or regions because of differences in medical settings.

The discussions held at the August 2021 advisory board meeting highlighted the similarities and differences in follow-up methods for asymptomatic *TTR* gene mutation carriers between Japan, Brazil, and Portugal (Table 2). In all three countries, follow-up before the disease onset is initiated in individuals originating from kindred with early-onset disease when they test positive for pathogenic mutations; in those originating from kindred with late-onset disease, follow-up is initiated 10 years before the predicted age of onset regardless of genotype (it should be noted that the age at onset may differ between the affected parent and child, especially in mother-son pairs [42]). Generally, referral centers in Brazil and Portugal actively employ noninvasive tests to monitor patients at the asymptomatic stage [11]. In contrast, Japanese referral centers perform tissue biopsies in addition to noninvasive tests for asymptomatic phase monitoring [7]. Moreover, in Japan, follow-up strategies differ slightly between individuals at risk of early-onset disease versus late-onset disease. For individuals at risk of early-onset disease, annual tissue biopsy is scheduled for the early detection of amyloid deposits and early diagnosis. For those at risk of late-onset disease, annual biopsy is not necessary but can be scheduled at the physician’s discretion (Table 2).

**Table 2:** Follow-up methods for *TTR* gene mutation carriers in Japan, Brazil, and Portugal

	Japan	Brazil and Portugal
Timing to initiate follow-up before the disease onset	Early-onset: when the genetic test reveals a positive result Late-onset: 10 years prior to the predicted age of onset (predicted based on the family history)^a^, annually after a positive genetic test	
Methods to follow-up before the disease onset	Early-onset: annual noninvasive tests, annual biopsy Late-onset: annual noninvasive tests (frequency can be increased at the physician’s discretion). Biopsy can be scheduled at the physician’s discretion	Annual noninvasive tests (frequency can be increased at the physician’s discretion)
Position of noninvasive tests and biopsies in diagnosis	Auxiliary diagnosis by noninvasive tests such as scintigraphy is useful, but the final definitive diagnosis is made by biopsy	Bone scintigraphy grade 2 or 3 sometimes replaces biopsy in late-onset cases with myocardiopathy
Main biopsy sites	Early-onset: abdominal fat (if not available, other less invasive sites can be selected) Late-onset: abdominal skin	Salivary glands, skin
Purpose of biopsy	To detect amyloid deposits before the symptom onset To establish a definitive diagnosis	To establish a definitive diagnosis (if the result is negative and the suspected diagnosis remains, another biopsy is scheduled after 3–6 months)
Types of noninvasive tests	Medical interview (sensation, movement, autonomic nerve function [orthostatic hypotension, gastrointestinal symptoms, and dysuria], weight loss, heart failure, arrhythmia, and ocular symptoms), physical examination (neurological, autonomic, cardiac, gastrointestinal, and ocular symptoms), blood tests (BNP or NT-proBNP, troponin T, transthyretin, albumin, creatinine, TSH, free T3, and free T4), renal function (eGFR and urinary microalbumin), blood pressure, cardiac evaluation (ECG [R-R interval]), Holter ECG, echocardiography, and ^99m^Tc-PYP myocardial scintigraphy), and ophthalmologic examination	Clinical evaluation (NIS, spine vs orthostatic blood pressure, and BMI), neurophysiology tests (nerve conduction study, sudomotor test [Sudoscan™], HRDB or heart rate variability, and QST), blood/urine biomarker tests (NT-proBNP, troponin, and others), and cardiac evaluation (^99m^Tc-DPD myocardial scintigraphy, MRI, echocardiography, and ECG)
References	[7]	[11]

The data were based on the collation of discussions from a medical advisory board meeting attended by amyloidosis specialists in Japan, Brazil, and Portugal. “Early-onset” represents individuals originating from kindred with early-onset disease, and “late-onset” represents those originating from kindred with late-onset disease

^a^The age at onset may differ between the affected parent and child, especially in mother-son pairs [42]

^*99m*^*Tc-DPD* ^99m^technetium-3,3-diphosphono-1,2-propanodicarboxylic acid, ^*99m*^*Tc-PYP* ^99m^technetium-pyrophosphate, *BMI* body mass index, *BNP* brain natriuretic peptide, *ECG* electrocardiography, *eGFR* estimated glomerular filtration rate, *HRDB* heart rate response to deep breathing, *MRI* magnetic resonance imaging, *NIS* Neuropathy Impairment Score, *NT-proBNP* N-terminal pro-brain natriuretic peptide, *QST* quantitative sensory testing, *T3* triiodothyronine, *T4* thyroxine, *TSH* thyroid-stimulating hormone, TTR transthyretin

#### Biopsy

As ATTRv amyloidosis is characterized by the deposition of transthyretin amyloid fibrils in organs and tissues, tissue biopsy using Congo red staining is commonly used for diagnosis, in conjunction with amyloid typing and genotyping [1, 10]. Commonly used biopsy sites include salivary glands, abdominal fat pads, skin, and gastrointestinal tract [43–46]. However, the invasive nature of tissue biopsies poses a substantial burden on patients undergoing the procedure. Moreover, diagnostic accuracy may vary depending on the biopsy site. Based on published literature, the sensitivity ranged from 73% for subcutaneous tissue to 91% for abdominal fat [43–45, 47]. In individuals at risk for the disease with cardiomyopathy, endomyocardial biopsy is also a good option. The sensitivity of endomyocardial biopsy is 100%, but this technique is highly invasive and needs to be performed in specialized centers and by skilled operators [47]. The specificity was reported to be 99% for abdominal fat biopsy [44] and 100% for subcutaneous abdominal fat aspiration [45]. Of note, the variability in the sensitivity of biopsies remains a controversial issue because the underlying data have been derived from multiple studies conducted in diverse settings.

In Brazil and Portugal, biopsies of the salivary glands and skin are used to establish a definitive diagnosis. In these countries, grade 2 or 3 bone scintigraphy sometimes replaces biopsy in late-onset cases with myocardiopathy. In rare cases, a positive biopsy may not be confirmed in the first years of disease, but a diagnosis can be made if other causes of neuropathy and myocardiopathy are excluded. In contrast, Japanese amyloidosis specialists commonly perform abdominal fat or abdominal skin biopsies because of their suitability for annual follow-ups; an auxiliary diagnosis by noninvasive tests such as scintigraphy is useful, but the final definitive diagnosis is made by biopsy (Table 2). Theoretically, amyloid deposition begins before the onset of signs and symptoms of amyloidosis, indicating the usefulness of periodic biopsy monitoring at asymptomatic stages [7, 48]. Periodic biopsy of asymptomatic *TTR* gene mutation carriers has been shown to help detect amyloid deposits earlier than the manifestation of clinical symptoms, although the number of patients assessed in the study was limited [7].

The utility of biopsy is discussed based on the experience in Japan, Portugal, and Brazil. For an accurate diagnosis of ATTRv amyloidosis using this technique, involvement of well-trained pathologists and amyloidosis specialists is essential. Diagnostic methods may be customized based on the medical settings of each country.

#### Noninvasive tests and examinations

Several noninvasive or less invasive tests and examinations have been introduced to capture the early signs of ATTRv amyloidosis. Such tests and examinations are performed at referral centers in Japan, Brazil, and Portugal to manage *TTR* gene mutation carriers (Table 2). The types of noninvasive tests and examinations used in Japan and the other two countries differ slightly. For example, nerve conduction studies, sudomotor tests, heart rate tests, and quantitative sensory testing (QST) [49–53] are more commonly performed in Brazil and Portugal than in Japan. The details and clinical implementations of noninvasive techniques for monitoring ATTRv amyloidosis are discussed in the next section (Management of ATTRv amyloidosis).

No published studies have directly compared biopsy and noninvasive testing as a measure to capture the early signs of ATTRv amyloidosis. However, case series reported from Japan may partly explain the utility of biopsy in detecting disease onset earlier than the manifestation of clinical symptoms [7].

### Management of ATTRv amyloidosis

#### The critical roles of Amyloidosis centers and referral networks

**Fig. 2:**
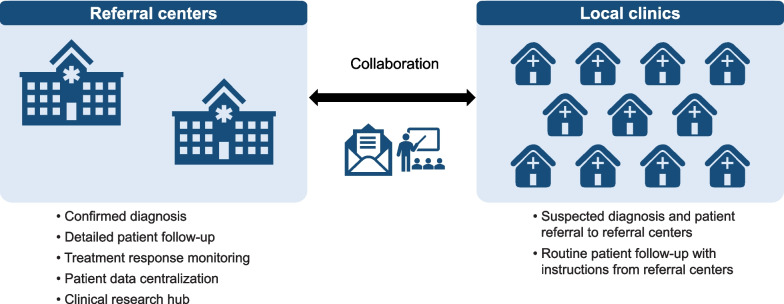
Model for a referral network in the post-onset management of ATTRv amyloidosis. Collaboration between referral centers and local clinics can be achieved by knowledge dissemination, patient referral, or other forms. *ATTRv* hereditary transthyretin

In the US, Japan, Brazil, and many European countries, referral centers specializing in amyloidosis have played critical roles in the diagnosis, follow-up, and treatment of patients, as well as in conducting clinical research for amyloidosis [6, 37, 54–60]. As demonstrated in the French FAP Network, which consists of a coordinating center in Paris and specialized rare neuromuscular disease centers located across the country [36], a referral network (Fig. 2) is crucial for the appropriate management of *TTR* gene mutation carriers and patients with ATTRv amyloidosis [61, 62]. In some countries, establishment of a referral network or amyloidosis center of excellence (CoE) may not be feasible; in such cases, treatment of patients with ATTRv should be considered a multidisciplinary team approach.

To establish an amyloidosis CoE and a robust referral network within a country, a report from Sofia, Bulgaria, can be referred to as a case study [60]. According to this report, the formulation of a multidisciplinary team was the first step in establishing a CoE, which involved a range of experts such as neurologists, ophthalmologists, cardiologists, gastroenterologists, nephrologists, geneticists, physiotherapists, psychologists, pathologists, and nurses. The CoE in Sofia plays a significant role in the education of staff at other referral centers and local hospitals, as well as in the collaboration with local patient advocacy groups [60].

#### Treatment options

Because transthyretin is mainly produced in the liver, liver transplantation has been the standard treatment for ATTRv amyloidosis since the 1990s [1, 63]. However, the number of liver transplantation cases for ATTRv amyloidosis has declined in the past several years owing to the introduction of pharmacotherapeutic agents [16]. Moreover, the survival of patients who underwent this procedure is not always favorable as anticipated [64, 65]. Disease-modifying treatments (DMTs) such as tafamidis meglumine (transthyretin stabilizer), patisiran, vutrisiran (small-interfering RNA), and inotersen (antisense oligonucleotide) have been approved for the treatment of ATTRv amyloidosis by multiple regulatory agencies [63, 66]. The approved dose of tafamidis for patients with the polyneuropathy phenotype (ATTRv amyloidosis with polyneuropathy) is 20 mg daily as tafamidis meglumine, and this drug is also approved for the treatment of transthyretin amyloid cardiomyopathy at a higher dose (80 mg daily as tafamidis meglumine or 61 mg daily as tafamidis) in many countries [16]. Diflunisal, an oral non-steroidal anti-inflammatory drug, has demonstrated efficacy in delaying neuropathy progression and improving the quality of life in patients with ATTRv amyloidosis [63]; however, its clinical implementation is currently restricted to off-label use [16]. The introduction of these pharmacotherapeutic options, which are most effective in patients who are the best candidates for liver transplantation (i.e., young, early-stage patients with the Val30Met mutation), has drastically reduced the role of liver transplantation in the treatment of ATTR amyloidosis [16].

Patient access to ATTRv amyloidosis treatment varies by country. For example, in Japan, liver transplantation, tafamidis meglumine, and patisiran are available [67], and vutrisiran was approved in September 2022 [68]. In Brazil, liver transplantation and tafamidis meglumine are the only available treatment options [58], whereas liver transplantation, tafamidis meglumine, patisiran, and inotersen are all available in Portugal (Teresa Coelho, personal communication). In Italy, diflunisal is among the preferred treatment options, in addition to liver transplantation and DMTs [55]. Off-label diflunisal is a common treatment choice in Sweden [69]. In China, tafamidis meglumine was approved in 2020 for the treatment of ATTRv amyloidosis [38].

#### Proposal for a set of practical assessments for routine clinical use

**Fig. 3:**
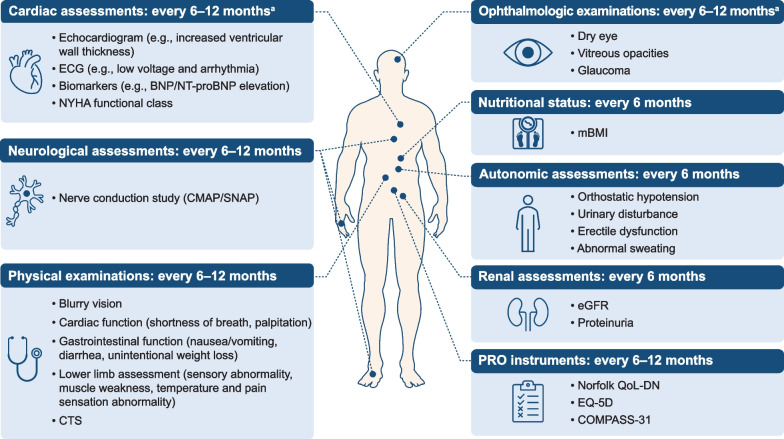
Set of practical tests and examinations for monitoring ATTRv amyloidosis progression. ^a^Cardiac and ophthalmologic assessments can be scheduled more frequently when any apparent symptoms are observed. *ATTRv* hereditary transthyretin, *BNP* brain natriuretic peptide, *CMAP* compound muscle action potential, *COMPASS-31* Composite Autonomic Symptom Score 31, *CTS* carpal tunnel syndrome, *ECG* electrocardiography, *eGFR* estimated glomerular filtration rate, *EQ-5D* EuroQol 5-dimension, *mBMI* modified body mass index, *NT-proBNP* N-terminal pro-brain natriuretic peptide, *NYHA* New York Heart Association, *QoL-DN* Quality of Life-Diabetic Neuropathy, *SNAP* sensory nerve action potential

In real-world clinical scenarios, neurologists treating patients with ATTRv amyloidosis often need to select the most suitable tests and examinations depending on their clinical experience and feasibility of each medical procedure. To aid in the selection of tests and examinations by amyloidosis specialists and general neurologists, a set of practical and convenient assessments for monitoring ATTRv amyloidosis progression (Fig. 3) would be useful. This set consists of assessments of patient’s nutritional status, autonomic function, and renal function scheduled every 6 months and cardiac function assessments, ophthalmologic examinations, and neurological assessments scheduled every 6 to 12 months. The cardiac and ophthalmologic assessments can be scheduled more frequently when any apparent symptoms are observed (Fig. 3). Some tests and examinations may be performed at referral centers or by ophthalmologists, internal medicine specialists, or cardiology specialists.

#### Noninvasive tests and examinations for ATTRv amyloidosis progression monitoring

Various noninvasive tests and examinations can be used to monitor disease progression in patients with ATTRv amyloidosis. In addition, individual treatment responses should be monitored as part of follow-up visits after disease onset because the response to DMTs may differ among patients [70, 71]. The tests and examinations, indices to detect disease progression, and feasibility of the tests and examinations to be performed in neurology clinics are summarized in Table 3.

**Table 3:** Noninvasive tests and examinations applicable to the monitoring of ATTRv amyloidosis progression

Type of noninvasive techniques	Details	Indices to detect disease progression	Feasibility to perform in neurology clinics^a^	References
Peripheral neuropathy assessments				
Items necessary to suspect disease onset	Upper limb numbness, pain in extremities, dissociated sensory disturbance	–	+ + +	[1]
Nerve conduction study	Measure the amplitude and velocity of sural and peroneal nerve conduction at left, right, or both sides of the body	SNAP amplitude ≤ 19 μV, sensory nerve conduction velocity ≤ 50 m/s, CMAP amplitude ≤ 16 mV, or motor nerve conduction velocity ≤ 51 m/s 50% reduction in CMAP amplitude within the normal range Reduction of CMAP or SNAP below the lower limit of normal range	+ +	[49]
QST	Evaluate sensory loss (hypesthesia, hypoalgesia) and gain (hyperalgesia, allodynia) for thermal and mechanical stimuli	–	+	[50]
NIS	Assess the degree of neuropathic symptoms (muscle weakness, reflexes, and sensation at specific sites) as a composite score	7- to 16-point increase in the total score	+	[16]
NIS-LL	Subset of the NIS specific to neuropathy in the lower limbs	≥ 2-point worsening in the total score	+	[73]
Autonomic function assessments				
Items necessary to suspect disease onset	Urinary disturbance, erectile dysfunction, orthostatic intolerance, diarrhea, constipation, alternating episodes of diarrhea and constipation, persistent nausea and vomiting, orthostatic hypotension, abnormal metaiodobenzylguanidine myocardial scans	–	+ + +	[1]
Supine versus orthostatic blood pressure	Assess orthostatic hypotension	≥ 20-mmHg decrease in SBP or ≥ 10-mmHg decrease in DBP after standing up from a sitting or supine position	+ + +	[82]
Sudomotor test (Sudoscan™)	Quantify sudomotor function through local conductance using chloride in the sweat	Feet ESC ≤ 66 µS	+	[51]
HRDB, heart rate variability	Detect cardiac autonomic dysfunction through a paced breath test	–	+	[52]
Cardiac symptom assessments				
Items necessary to suspect disease onset	Edema, conduction disorders with syncope, ventricular wall thickness and/or low voltage, elevated plasma BNP or NT-proBNP levels, abnormal cardiac accumulation in ^99m^technetium-labeled tracer scintigraphy	–	+ + +	[1]
Echocardiography	Monitor cardiac involvement	Increased ventricular wall thickness (> 12 mm)	+ +	[83]
ECG	Monitor cardiac involvement	Low QRS voltage, conduction disturbance, arrhythmia	+ +	[83]
BNP, NT-proBNP, troponin T, troponin I	Monitor cardiac function	Elevation in BNP, NT-proBNP, troponin T, or troponin I	+ +	[84]
NYHA functional classification	Classify the severity of heart failure symptoms (class I, II, III, or IV)	Class II or above	+ +	[83]
Cardiac MRI	Monitor cardiac involvement	Late gadolinium enhancement	+	[83]
Myocardial scintigraphy using ^99m^technetium-labeled tracers	Monitor cardiac amyloidosis	Grade 2 (moderate uptake, equal to rib uptake) or grade 3 (high uptake, greater than rib uptake) myocardial uptake with planar imaging	+	[85]
CTS				
Phalen’s test	Monitor the development of CTS by putting pressure on the carpal tunnel	Exacerbation of dysesthesia after keeping the wrists flexed for 1 min	+ +	[67]
Reverse Phalen’s test	Monitor the development of CTS by putting pressure on the carpal tunnel	Exacerbation of dysesthesia after keeping the wrists extended for 1 min	+ +	[67]
Tinel’s sign test	Monitor the development of CTS by tapping the carpal tunnel with a hammer	Tingling pain	+ +	[67]
Nerve conduction study	Measure the amplitude and velocity of median nerve conduction at left, right, or both sides of the body	Sensory nerve conduction velocity of the median nerve across the carpal tunnel < 45 m/s; difference between the latencies of sensory potentials of median nerve determined at the fourth finger after equidistant stimulation of ulnar and median nerve > 0.5 ms; or distal motor latency > 4.4 ms for a stimulus applied 8 cm away from active motor electrode	+ +	[87]
Nutritional status				
Items necessary to suspect disease onset	Unexplained weight loss	–	+ + +	[1]
mBMI	Monitor nutritional status and detect unexplained weight loss	mBMI < 1000	+ + +	[76]
Renal assessments				
eGFR and urinary protein	Monitor renal dysfunction due to amyloidosis	eGFR < 60 mL/min/1.73 m^2^, abnormal urinary protein excretion (> 150 mg/24 h), or albuminuria (> 30 mg/24 h or mg/g creatinine)	+ + +	[4]
PRO instruments				
Norfolk QoL-DN	Assess neuropathy-specific changes in patients’ quality of life	Norfolk QoL-DN score of ≥ 48	+ +	[77–80]
EQ-5D	Measure general health status	Utility value of ≤ 0.5	+ +	[92]
COMPASS-31	Assess patients’ autonomic functions	Total COMPASS-31 score of ≥ 30	+ +	[78]

^a^Feasibility to perform in neurology clinics was assessed as + (difficult to perform), +  + (intermediate), or +  +  + (easy to perform) based on the authors’ clinical experience

*ATTRv* hereditary transthyretin, *BMI* body mass index, *BNP* brain natriuretic peptide, *CMAP* compound muscle action potential, *COMPASS-31* Composite Autonomic Symptom Score 31, *CTS* carpal tunnel syndrome, *DBP* diastolic blood pressure, *ECG* electrocardiography, *eGFR* estimated glomerular filtration rate, *ESC* electrochemical skin conductance, *EQ-5D* EuroQol 5-dimension, *HRDB* heart rate response to deep breathing, *mBMI* modified body mass index, *MRI* magnetic resonance imaging, *NIS* Neuropathy Impairment Score, *NIS-LL* Neuropathy Impairment Score in the Lower Limbs, *NT-proBNP* N-terminal pro-brain natriuretic peptide, *NYHA* New York Heart Association, *PRO* patient-reported outcome, *QoL-DN* Quality of Life-Diabetic Neuropathy, *QST* quantitative sensory testing, *SBP* systolic blood pressure, *SNAP* sensory nerve action potential

#### Peripheral neuropathy assessments

For routine patient assessments, it is advisable to perform physical examinations focusing on peripheral neuropathy in the lower limbs. Sensory abnormalities, muscle weakness, and abnormalities in temperature and pain sensation may suggest disease progression in patients with ATTRv amyloidosis (Fig. 3).

In terms of noninvasive tests to assess peripheral neuropathy, the THAOS Registry has captured nerve conduction study data based on local/regional clinical practice, which measure the amplitude and velocity of the sural and peroneal nerves at the left, right, or both sides of the body [49]. According to the analyses of nerve conduction data captured in the THAOS Registry, the mean sural sensory nerve action potential (SNAP) was 19.2 μV in amplitude and 50.7 m/s in velocity; the mean peroneal nerve compound muscle action potential (CMAP) was 16.4 mV in amplitude and 51.7 m/s in velocity [49]; these data may be helpful for monitoring ATTRv amyloidosis progression in routine clinical settings. The SNAP and CMAP amplitude and velocity values decreased with advanced disease stage, indicating their utility in monitoring ATTRv amyloidosis progression [49].

QST assesses sensory loss and gain in response to thermal and mechanical stimuli [50]. Research findings using QST have suggested that patients with ATTRv amyloidosis have impaired cold perception and mechanical hyperalgesia in their hands [50]. However, the instruments and methodological approaches for QST are yet to be standardized; therefore, its suitability in routine patient follow-ups and the establishment of universally applicable cutoff scores warrant further investigation [14]. Moreover, based on our experience, QST may be a time-consuming procedure and thus may not always be used in routine clinical practice.

The Neuropathy Impairment Score (NIS) is a composite score designed to quantify neuropathic muscle impairments [72]. The Neuropathy Impairment Score-lower limb (NIS-LL) is a subset of NIS that assesses neuropathic impairments in the lower limbs [72]. Both scales have been used successfully to detect neuropathy progression in patients with ATTRv amyloidosis in multiple clinical studies [72, 73] and in clinical settings [74–76]. A 7- to 16-point increase in the NIS total score [16] or ≥ 2-point worsening of the NIS-LL total score [73] can be an indicator of disease progression. However, the NIS and NIS-LL are subjective scales assessed by physicians and may cause variability in the results; therefore, the involvement of trained and experienced neurologists is crucial to maximize benefit from using these scales [72]. Updated versions of the NIS, such as the Neuropathy Impairment Score + 7 (NIS + 7) and the modified NIS + 7 (mNIS + 7), have been used in several clinical trials [77–80] but may not be suitable for routine clinical use because of their complexity. Other scales to measure the overall disease status, such as the familial amyloid polyneuropathy (FAP) stage and polyneuropathy disability (PND) score, can be used but may not be sufficiently sensitive [81].

#### Autonomic function assessments

Orthostatic hypotension can be detected by measuring the supine versus orthostatic blood pressure. ATTRv amyloidosis progression should be suspected when a patient shows a ≥ 20-mmHg decrease in systolic blood pressure or ≥ 10-mmHg decrease in diastolic blood pressure 3 min after standing up from a sitting or supine position [82]. Orthostatic hypotension and other autonomic dysfunctions such as urinary disturbance, erectile dysfunction, and abnormal sweating (Fig. 3) can be assessed primarily through medical examinations.

The sudomotor test was developed as a noninvasive neurophysiological technique to quantify sudomotor function through local electrochemical skin conductance using sweat chloride [51]. A specifically designed device (Sudoscan™) is required to perform this test. This technique can detect autonomic dysfunction in patients with ATTRv amyloidosis; when using the feet, the electrochemical skin conductance cutoff value is reportedly 66 µS, with a sensitivity and specificity of 76% and 85%, respectively [51].

The heart rate response to deep breathing or heart rate variability test is an indicator developed to detect cardiac autonomic dysfunction, but the results may be heavily affected by the presence of arrhythmias [52]. Therefore, this test should be applied in conjunction with careful review of patients’ electrocardiography (ECG) data by an expert [52].

#### Cardiac symptom assessments

Echocardiography and ECG are helpful for monitoring the levels of cardiac amyloidosis in routine clinical settings [83]. Patients with ATTRv amyloidosis progression may present with increased ventricular wall thickness on echocardiography or exhibit characteristic ECG findings such as low QRS voltage, conduction disturbance, and arrhythmia [83]. Results from blood tests to monitor cardiac function (e.g., brain natriuretic peptide, N-terminal pro-brain natriuretic peptide, troponin T, and troponin I) [84] are also convenient biomarkers. To evaluate the degree of cardiac symptoms in each patient, the New York Heart Association functional classification, which classifies the severity of heart failure symptoms as Class I to Class IV, is useful [83].

During routine patient follow-up, physical examinations should include cardiac function assessments. Cardiac findings, such as shortness of breath and palpitations, can be signs of disease progression in patients with ATTRv amyloidosis (Fig. 3).

Cardiac magnetic resonance imaging and myocardial scintigraphy using ^99m^technetium-labeled tracers are also powerful diagnostic modalities for monitoring disease progression [83, 85]. However, patient assessment using these modalities may require the involvement of centers and experts specializing in the diagnosis of ATTRv amyloidosis. Moreover, the diagnostic sensitivity of ^99m^technetium-labeled tracer scintigraphy is low for some *TTR* genotypes such as Ser77Tyr (p.Ser98Tyr) and Phe64Leu (p.Phe84Leu) [85], as well as early-onset Val30Met mutation [86].

#### CTS

Patients with ATTRv amyloidosis commonly develop CTS as initial symptom manifestation [5, 6]. These findings underpin the usefulness of CTS assessments for monitoring disease progression in routine neurological practice. CTS can present as thenar muscle atrophy, numbness in the thumb to the thumb side of the ring finger, difficulties in fine motor skills such as buttoning up, or inability to create a neat circle (perfect O-sign) with the thumb and index finger [67, 85]. In addition, Phalen’s test, reverse Phalen’s test, and Tinel’s sign test are helpful for routine clinical use. Phalen’s test examines the exacerbation of dysesthesia due to increased carpal tunnel pressure by keeping the wrists flexed for 1 min. Reverse Phalen’s test examines the same sign as Phalen’s test using extended wrists. Tingling pain observed when the carpal tunnel is tapped with a hammer is characterized by Tinel’s sign and is a finding suggestive of CTS [67]. CTS can be also identified using nerve conduction studies [87].

#### Nutritional status

Approximately 30% of patients with ATTRv amyloidosis present with unexplained weight loss [88, 89]. Body mass index (BMI) and modified BMI (mBMI) are helpful in monitoring wasting and autonomic gastrointestinal dysfunction in patients with ATTRv amyloidosis [76]. The mBMI, which is calculated by multiplying the BMI (kg/m^2^) by the serum albumin level (g/L), is recommended over the conventional BMI because it corrects the effect of edema resulting from low serum albumin levels [76]. An observational, cross-sectional, single-center study conducted in Portugal reported a statistically significant decrease in the mean mBMI with advanced ATTRv amyloidosis stage, from 1199.0 in healthy adults to 759.7 in patients with the Coutinho stage 3 disease (wheelchair bound or bedridden) [76].

In addition, disease progression should be suspected in patients presenting with findings suggestive of gastrointestinal dysfunction such as nausea, vomiting, diarrhea, and unintentional weight loss (Fig. 3).

#### Renal assessments

Estimated glomerular filtration rate (eGFR) and renal biomarkers such as urinary proteins can be used for assessing renal function in routine clinical settings (Fig. 3). According to research findings derived from patient data review in an Italian center, among 46 patients with ATTRv amyloidosis, 15% showed reduced eGFR (< 60 mL/min/1.73 m^2^) and 22% showed abnormal urinary protein excretion (> 150 mg/24 h) and/or albuminuria (> 30 mg/24 h or mg/g creatinine) [4].

#### Ophthalmologic examinations

Ocular involvement is another indicator of ATTRv amyloidosis [3]. Typical ophthalmologic findings associated with ATTRv amyloidosis include vitreous opacity and glaucoma [3], and patients may complain of symptoms such as dry eye and bleariness (Fig. 3).

#### Patient-reported outcomes

Disease progression in patients with ATTRv amyloidosis can be monitored using patient-reported outcome (PRO) instruments (Fig. 3, Table 3). Notably, in non-English-speaking countries and regions, the availability and validity of the PRO questionnaire translated into the local language need to be checked before its clinical implementation.

The Norfolk Quality of Life-Diabetic Neuropathy (QoL-DN) questionnaire consists of 47 items evaluating neuropathic symptoms in the feet, legs, hands, and arms; complications; activities of daily living; and generic health status [90]. It has been used in clinical trials for ATTRv amyloidosis [77–80] and has been suggested to be a reliable measure of disease progression [76]. The approximate baseline Norfolk QoL-DN score reported for previous clinical trials of patients with ATTRv amyloidosis ranged from 47 to 60 [77–80].

The EuroQol 5-dimension (EQ-5D) is a self-administered questionnaire developed by the EuroQol group to assess an individual’s generic health status [91]. According to a multi-institutional, longitudinal, prospective, observational study conducted in Portugal, the estimated EQ-5D utility value for a patient with ATTRv amyloidosis was 0.51, which was 0.27 point lower than that for the general population (0.78) [92].

The Composite Autonomic Symptom Score 31 (COMPASS-31) is a 31-item self-administered questionnaire developed for the quantitative assessment of patients’ autonomic symptoms [93]. A total COMPASS-31 score of 30, a score recorded at baseline in a phase 3 clinical trial of patisiran, can be an indicator of ATTRv amyloidosis progression [77, 78].

Furthermore, several PRO instruments have been used for the self-assessment of the quality of life of patients with ATTRv amyloidosis, such as the Kansas City Cardiomyopathy Questionnaire, Patient General Assessment, and 36-Item Short Form Survey [94]. However, their suitability for disease progression markers, strengths, limitations, and feasibility to be performed in routine clinical settings should be evaluated thoroughly before introduction into routine neurology care.

### Future perspectives

Amyloidosis specialists worldwide have actively developed expert recommendations to manage *TTR* gene mutation carriers and improve the early diagnosis of ATTRv amyloidosis [10–12, 27, 28, 41, 62, 95, 96]. Moreover, proposals for the minimum set of assessments provided in the ISA guidelines [16] and the set of assessments discussed in this article are expected to facilitate appropriate patient management after the onset of ATTRv amyloidosis.

However, several challenges remain unaddressed. For example, the future role of biopsy and its significance in asymptomatic stage management should be discussed in greater detail. Additionally, a robust psychological care process to alleviate the psychological burden of the disease is crucial. Such a process would be beneficial not only to at-risk individuals or patients developing ATTRv amyloidosis but also to their relatives and caregivers [97]. Regarding the monitoring of ATTRv amyloidosis progression after disease onset, novel tests and assessments are being explored, such as urinary transthyretin [98], intraepidermal nerve fiber density [46, 99], nerve ultrasound [100], metabolomics analysis [101], serum inflammation markers [102], corneal confocal microscopy and electroretinogram [103], plasma neurofilament light chain [104], and gait parameter analysis [105]. Further clinical research is warranted to establish the diagnostic accuracy of these assessments and investigate their feasibility in routine neurological practice. An international consensus on genetic counseling protocols, patient educational programs, simplified questionnaires or disease scoring systems, and validated clinical markers for treatment responses would provide adequate guidance for routine clinical use.

### Limitations

This review has some limitations. First, most epidemiology data of ATTRv amyloidosis reported by the THAOS Registry are derived from the countries where the Val30Met mutation is the predominant genotype. Therefore, the generalizability of the findings to other countries may be limited. Moreover, the diagnostic methods and indices to detect disease progression discussed in the current review are based on the experience in Japan, Brazil, and Portugal and may need adaptation before use in other countries where non-Val30Met mutations are predominant.

## Conclusions

For the appropriate pre- and post-symptom onset testing for ATTRv amyloidosis, it is important to capture the signs of disease onset in asymptomatic *TTR* gene mutation carriers and monitor disease progression after diagnosis. Our discussion at the advisory board meeting revealed that the methodology used to monitor *TTR* gene mutation carriers differs among countries in terms of the use of noninvasive tests and tissue biopsies. For monitoring ATTRv amyloidosis progression, developing a set of tests and examinations that can be applied in diverse medical settings could allow effective patient follow-up. This literature could serve as a guide for neurologists around the world in choosing the tests and examinations based on their feasibility to best manage *TTR* gene mutation carriers and patients with ATTRv amyloidosis. We hope that this review will assist neurologists in better understanding the management of ATTRv amyloidosis in routine practice.

## Data Availability

Data sharing is not applicable to this article as no datasets were generated or analyzed during the current study.

## Abbreviations

ATTRv: Hereditary transthyretin
BMI: Body mass index
CMAP: Compound muscle action potential
CoE: Center of excellence
COMPASS-31: Composite autonomic symptom score 31
CTS: Carpal tunnel syndrome
DMT: Disease-modifying treatment
ECG: Electrocardiography
eGFR: Estimated glomerular filtration rate
EQ-5D: EuroQol 5-dimension
FAP: Familial amyloid polyneuropathy
ISA: International Society of Amyloidosis
mBMI: Modified body mass index
mNIS + 7: Modified neuropathy impairment score + 7
NIS: Neuropathy impairment score
NIS-LL: Neuropathy impairment score-lower limb
NIS + 7: Neuropathy impairment score + 7
PND: Polyneuropathy disability
PRO: Patient-reported outcome
QoL-DN: Quality of life-diabetic neuropathy
QST: Quantitative sensory testing
SNAP: Sensory nerve action potential
THAOS: Transthyretin amyloidosis outcomes survey
*TTR*: Transthyretin
US: United States
